# Endothelial Progenitor Cell Dysfunction in Polycystic Ovary Syndrome: Implications for The Genesis of Cardiovascular Diseases

**Published:** 2013-03-03

**Authors:** Yu-Hsun Kao, Wan-Chun Chiu, Ming-I Hsu, Yi-Jen Chen

**Affiliations:** 1 Department of Medical Education and Research, Wan Fang Hospital, Taipei Medical University, Taipei, Taiwan; 2 Graduate Institute of Clinical Medicine, College of Medicine, Taipei Medical University, Taipei, Taiwan; 3 School of Nutrition and Health Sciences, Taipei Medical University, Taipei, Taiwan; 4 Department of Obstetrics and Gynecology, Wan Fang Hospital, Taipei Medical University, Taipei, Taiwan; 5 Division of Cardiovascular Medicine, Department of Internal Medicine, Wan Fang Hospital, Taipei Medical University, Taipei, Taiwan

**Keywords:** Polycystic Ovary Syndrome, Progenitor Cells, Cardiovascular Disease, Endothelial

## Abstract

Polycystic ovary syndrome (PCOS), the most common endocrine disorder affecting women of reproductive age, is characterized by hyperandrogenism and insulin resistance. Women with PCOS have a higher risk for cardiovascular diseases (CVDs) and endothelial dysfunction. The mechanisms underlying these risks are unclear. Human peripheral blood contains circulating endothelial progenitor cells (EPCs) derived from bone marrow that have the ability to proliferate and differentiate into mature endothelial cells, which may contribute to vessel homeostasis and repair. PCOS is associated with insulin resistance, hyperinsulinemia, and dyslipidemia, which may result in EPC dysfunction. In this review, we summarize the potential mechanisms of EPC dysfunction in PCOS, which possibly result in a higher genesis of CVDs in PCOS-affected subjects.

## Introduction

Polycystic ovary syndrome (PCOS) is the most common endocrine disorder in reproductive women. It is estimated that 5-11% of women of reproductive age have PCOS (1-4). One of the major diagnostic criteria of PCOS is chronic anovulation which leads to irregular menstruation, amenorrhea and infertility; the other diagnostic criterion is hyperandrogenism which leads to hirsutism, acne, and alopecia. Women with PCOS are at high risk for developing cardiovascular diseases (CVDs) (5-7) and exhibit endothelial dysfunction. Endothelial progenitor cells (EPCs) play an important role in the pathophysiology of CVDs. EPCs can home to sites of neovascularization and differentiate into endothelial cells in response to a variety of stimuli (8-10). PCOS is associated with hypertension, obesity, dyslipidemia, and insulin resistance, all of which may result in EPC dysfunction (11). Endothelial dysfunction has been observed in PCOS patients despite normal glycemia, lipidemia, and blood pressure, and without structural arterial impairment (12-16). In this review, we summarize the potential mechanisms of EPC dysfunction in PCOS, which can result in a higher genesis of CVD in PCOS-affected subjects.

### PCOS and CVDs

PCOS-affected women have a number of reproductive and metabolic abnormalities. Previous studies of PCOS women with body mass index (BMI)-matched controls have proposed several CVD risk factors related to PCOS (17, 18). PCOS is frequently associated with obesity, elevated blood pressure, and dyslipidemia (19, 20); all of which are important risk factors for CVDs. PCOS patients have increased non-traditional risk factors for CVDs, such as elevated homocysteine (21-23), C-reactive protein (24), plasminogen activator inhibitor-1 (25), and fibrinogen (26) levels. In addition, our previous study has found evidence of a widening QRS complex (a biomarker for heart failure) on electrocardiogram in PCOS patients (27).

Through a calcium score analysis, PCOS patients had increased prevalence of coronary artery disease (CAD) independent of BMI and age. Shroff et al. have reported a correlation between CAD and PCOS using coronary artery calcium and inflammatory markers (28). Therefore, PCOS is an important risk factor for CAD. PCOS patients also have an increased risk of cerebrovascular diseases (29). Increased carotid intimal-medial thickness and carotid atherosclerotic plaque index scores have been reported in PCOS patients (30, 31). Asymmetrical dimethyl-L-arginine (ADMA) is an endogenous nitric oxide synthase (NOS) inhibitor, which can induce atherosclerosis and serve as an independent marker for cardiovascular morbidity (32). PCOS women have elevated ADMA (33), which may induce endothelial dysfunction in these patients. The above findings suggest that CVD risk, as reflected by endothelial dysfunction, is increased in PCOS patients. Table 1 summarizes the clinical evidence of PCOS in CVDs.

### EPC dysfunction contributes to CVDs

EPCs play critical roles in endothelial function and the genesis of atherosclerosis (34, 35). Bone marrow-derived peripheral EPCs can home to sites of vessel growth, where they proliferate and differentiate into mature endothelial cells for neovascularization (10). Aging, diabetes, hypercholesterolemia, and stroke are associated with impaired neovascularization, which may be caused by EPC dysfunction (36, 37). Peripheral EPCs isolated from CAD patients are significantly declined, revealing an impaired migratory response (38). Similarly, decreased EPCs may result in a poor outcomes after ischemic stroke (39).

Circulating EPC numbers and function were significantly reduced in diabetic patients with peripheral artery disease (PAD), and the severity of carotid stenosis was negatively correlated with the EPC number in these patients (40). In addition, angiotensin II and oxidative stress possibly contribute to reduced EPC number and function through activation of the AT1a receptor (41). Therefore, EPCs significantly contribute to the pathophysiology of CVDs.

### Potential EPC disorders in PCOS

Endothelial dysfunction is a common finding in PCOS patients (13, 42). EPCs have been shown to play a critical role in regulating endothelial function (43-45). According to recent studies, PCOS patients have reduced EPC numbers and impaired EPC function along with increased central arterial stiffness. Our studies have reported the presence of hyperinsulinemia and insulin resistance in PCOS patients (46, 47), which may result in EPC dysfunction through increased reactive oxygen species and impaired insulin signaling (48). When EPCs from insulin-resistant Zucker fatty rats were exposed to tumor necrosis factor-α, there was increased apoptosis and decreased AKT phosphorylation in the EPCs, which suggested that inflammation could induce EPC dysfunction. In addition, our studies found that hyperglycemia significantly modulated peroxisome proliferator-activated receptor and cardiac inflammation (49, 50), which were effects that have been shown to impair EPC function (51). Since PCOS is associated with a hyper-inflammatory status, inflammation-related EPC dysfunction could contribute to increased CVDs in PCOS patients. According to Gallagher et al. diabetic mice have an approximately 50% reduction in circulating EPCs compared to non-diabetic controls (52). PCOS is frequently combined with obesity, which can induce inflammation and oxidative stress, thus resulting in (42) EPC dysfunction. Oxidized lowdensity lipoprotein has been shown to impair EPC migration and endothelial NOS. Therefore, dyslipidemia from PCOS can also produce EPC dysfunction.

The prevalence of insufficient vitamin D is higher in PCOS patients (53). Vitamin D dysregulation and deficiency is correlated with CVDs and affects EPCs (54, 55). Therefore, administration of vitamin D may have beneficial effects on CVD risk factors in PCOS patients (56-58). Accordingly, vitamin D deficiency may reduce the EPC number and function in PCOS patients as a result of developing CVDs. Various environmental chemical toxicants have also been implicated in endocrine disruption that may be associated with PCOS. PCOS patients have a higher blood level of bisphenol A (BPA), an estrogenic endocrine-disrupting chemical used to produce plastics (59). Since chemical toxicants increase CVDs and are known to affect EPCs (60, 61), it is possible that chemical toxicants may reduce the EPC number and function in PCOS patients, thus increasing CVDs.

**Table 1:** Clinical evidences of the cardiovascular risk in PCOS


Disease	Study design	Outcome

**CAD**	Compared coronary artery calcium in PCOS patients and healthy controls	A higher incidence of coronary artery calcium in PCOS patients (33%) than in controls (8%) (28).
		PCOS was associated with increase coronary artery calcium after adjusting for age, BMI, and menopausal status (62).
	Compared CAD risk factors between PCOS and healthy females	Increased BMI, total cholesterol, triglyceride, LDL, SBP, DBP, insulin, glucose, and HOMAIR (63).
	Compared cardiovascular outcomes in PCOS and healthy women	Increased cardiovascular events in PCOS patients compared to controls, with an odds ratio of 5.91 (64).
**Stroke**	Compared the carotid intimal-media thickness (IMT) by echography in PCOS patients and healthy controls	Increased IMT in PCOS patients (0.58 vs. 0.47 mm) than in healthy controls (31).
	Compared the carotid artery ultrasonographs of PCOS patients and controls	Higher prevalence of an abnormal carotid plaque index in PCOS patients than in controls (7.2 vs. 0.7%) (30).

BMI; Body-mass index, LDL; Low-density lipoprotein, SBP; Systolic blood pressure, DBP; Diastolic blood pressure, HOMAIR; Homeostasis model assessment of insulin resistance, CAD; Coronary artery disease and IMT; intimal-media thickness.

## Conclusion

**Fig 1:**
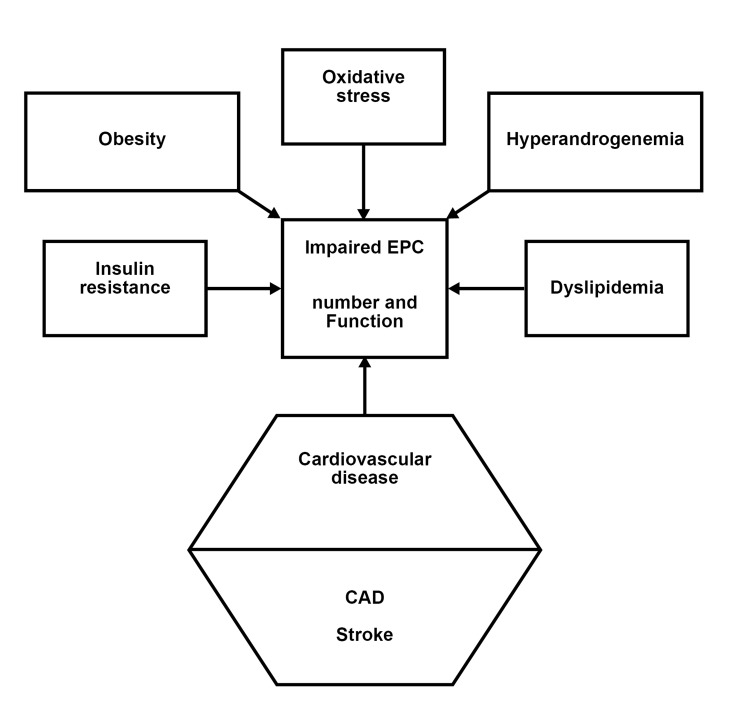
Mechanisms underlying endothelial progenitor cell (EPC) dysfunction in polycystic ovarian syndrome (PCOS) which contribute to cardiovascular disease.

PCOS is an independent marker of long-term cardiovascular risk and plays an important role in the pathophysiology of CVDs. EPCs maintain endothelial repaired capacity in mature blood vessels. Impaired EPC number and function will produce endothelial dysfunction and CVD progression (Fig 1). Therefore, EPC dysregulation may contribute to the genesis of CVDs in PCOS patients.
